# A Rapid, High-Resolution Chromatographic Method for Isolating Subpopulations of HEK293-derived Extracellular Vesicles

**DOI:** 10.7150/ntno.132261

**Published:** 2026-04-16

**Authors:** Raphael Ewonde Ewonde, William F. Pons, R. Kenneth Marcus

**Affiliations:** Department of Chemistry, Biosystems Research Complex, Clemson University, Clemson, SC 29634-0973, USA.

**Keywords:** Extracellular vesicles, polyester fibers, HEK293, subpopulation isolation, hydrophobic interaction chromatography, reproducibility, high-resolution method.

## Abstract

**Rationale:**

In recent years, significant advances have been made in understanding the basic underlying science of extracellular vesicles (EVs). This has opened multiple avenues for potential application, especially in areas such as biomarker discovery and drug delivery (vector) systems. Nonetheless, to achieve this, challenges such as the lack of reproducible isolation methods with high resolving power, especially for enriching subpopulations of EVs, need to be tackled. Here, we developed a hydrophobic interaction chromatography (HIC) method utilizing cost-effective polyester fiber columns for the isolation of EVs.

**Methods:**

Human embryonic kidney (HEK)-293 cells were cultured in shake flasks, centrifuged and filtered to remove cells and cell debris, respectively. The clarified media was injected onto capillary-channeled polymer (C-CP) fiber columns in HIC mode using step gradients. Alternative gradients that combine both positive and negative steps (termed a switchback gradient) during EV elution were scouted to improve the separation of partially resolved peaks. The optimized method was transferred to an analytical-scale column, and peaks were collected using an integrated fraction collector. For downstream characterization, fractionated peaks were buffer exchanged using Amicon filters with a 10 kDa molecular weight cut-off.

**Results:**

Polyester fiber columns, operated with both positive and negative step gradients during EV elution, yield baseline separation of three EV peaks within 12 minutes. Retention times show high repeatability (<0.33% RSD) and reproducibility (<1.3% RSD) across three column batches. Characterization of each peak fraction using nanoparticle tracking analysis (NTA) and nanoflow cytometry (nFCM) revealed similar trends in the size variation. The variation in the surface markers CD9 and CD81 among the collected fractions was confirmed by nFCM in the fluorescent detection mode, while intact double-layer and cup-shaped vesicles were observed in transmission electron microscopy (TEM) images.

**Conclusions:**

We demonstrated here, for the first time, a rapid chromatographic method for isolating and enriching EV subpopulations based on their chromatographic behavior which is reflective of their hydrophobicity (potentially a function of size or surface protein density) in a single unit operation. Three discrete size populations of EVs (based on NTA and nFCM sizing) were baseline separated within 12 minutes. Preliminary characterization of surface protein composition via nFCM showed significant differences among the isolated subpopulations.

## Introduction

Extracellular vesicles (EVs) are cell-derived nanovesicles with sizes ranging from 40 nm to 1000 nm, defined by a lipid bilayer membrane surrounding an aqueous lumen. The molecular content of EVs includes lipids, proteins, nucleic acids, metabolites, and other soluble mediators that can alter cellular processes 1, 2. This makes EVs highly heterogeneous, not only in their molecular composition, but also in terms of size and density. Notably, EVs in the lower size range (< 200 nm), generally termed exosomes, are known to possess several key attractive attributes, including biocompatibility and immune tolerance 3. deep tissue penetration,^4^ cellular uptake,^5^ and suitability for genetic manipulation 6. These intrinsic attributes have contributed to the growing interest in EVs in recent years, particularly for potential clinical applications and therapeutic development 7-10. However, limitations in separation and characterization methods, as well as functional studies, remain the major bottlenecks hindering the development of translational therapeutics.

While functional subpopulations exist between EVs from different sources, even EVs from the same source and cell type have been shown to possess different molecular composition in both physiological and pathological conditions. As such, heterogeneity analysis is crucial for elucidating the composition of various subpopulations and, therefore, the specific physiological functions 11. Assigning the functions of EVs to the bulk population hampers advances in practical applications, for instance, in therapeutics; the presence of subpopulations that lack the desired effect will result in reduced potency.

EVs can be isolated from a wide range of sources, such as biofluids (blood, urine, saliva), as well as cell culture, which is preferred for large-scale production 12. The human embryonic kidney (HEK)-293 cell line has been used for EV production, with the culturing conditions becoming well established 13, 14. This cell line offers several advantages, including a rapid growth rate, batch-to-batch reproducibility, and minimal growth requirements in suspension culture using chemically defined or serum-free media 2, 12, 13. A plethora of EV isolation methods exists, each with its own pros and cons 13, 15-17. Size and density-based methods, such as ultracentrifugation, size exclusion chromatography, and density gradient ultracentrifugation, are the most frequently used 18. However, these methods offer limited resolving power and often lead to co-isolation of contaminants having similar sizes and densities; hence, they cannot selectively enrich EV subpopulations. Therefore, there is a critical need for facile, high-resolution methods that can enrich subpopulations of EVs 19. This will enhance downstream characterization and ultimately facilitate an understanding of EV heterogeneity biology, as well as the discovery of EV-based biomarkers and therapeutics 12, 13, 19. The membrane of EVs is essentially decorated with surface proteins, conferring a degree of hydrophobicity that can be exploited for separation in hydrophobic interaction chromatography (HIC). In this regard, our group has extensively used HIC for isolation of EVs from diverse sources (milk, urine, cell culture media), applying polymer fibers as a stationary phase in capillary column format and spin-down tips 20-26. Here, we describe and demonstrate a novel, cost-effective “switchback gradient” method based on HIC for the high-resolution isolation of EV subpopulations from HEK293 cell cultures.

## Methods

### Chemicals and materials

Ammonium sulfate (≥99.0%), disodium hydrogen phosphate (≥99.0%), sodium dihydrogen phosphate (≥98.0%), sodium hydroxide, and Amicon Ultra-15 (10 kDa MWC) spin vials were purchased from Sigma-Aldrich (Spruce Street, MO, USA). HPLC-grade acetonitrile (ACN) was purchased from VWR Chemicals (Radnor, PA, USA), and Ultrapure water was produced in-house using an ELGA LabWater purification system (PURELAB flex, High Wycombe, UK). Carbon-coated 200 mesh grids and uranyl acetate were purchased from Electron Microscopy Sciences (Hatfield, PA, USA).

### Instrumentation and experimental conditions

HIC experiments were performed on a Vanquish Flex UHPLC system (Thermo Fisher Scientific, Sunnyvale, CA, USA), consisting of a quaternary pump F, a split sampler FG, a UV-Vis photodiode array detector VF equipped with a 13 µL flow cell, and an integral fraction collector FT. Viper MP53N tubing was used to connect the different UHPLC modules. Samples were maintained at 5 °C in the autosampler and fraction collector. The injection volume was set at 4 µL, and the flow rate was set at 0.5 mL min^-1^, unless stated otherwise. Optical absorbance data were recorded at a wavelength of 216 nm, at a sampling rate of 5 Hz and a response time of 1 s. Chromeleon software (version 7.3.2) was used for instrument control and data management.

### Mobile phase and sample preparation

The quaternary mobile phases (MPs) used were prepared using Ultrapure water and consisted of 2 M ammonium sulfate in 50 mM phosphate (MP-A), 1 M ammonium sulfate/ 10% ACN in 50 mM phosphate (MP-B), 35% ACN in 50 mM phosphate (MP-C), and 50 mM phosphate buffer (MP-D). The mobile phase pH was adjusted by adjusting the ratio of disodium hydrogen phosphate and sodium dihydrogen phosphate 27. The pH was adjusted to 7.0 by the addition of a 2 M sodium hydroxide solution. MPs were filtered through a 0.22 µm pore, 500 mL polyethersulfone (PES) vacuum filtration unit (VWR, Radnor, PA, USA).

The cell stocks used throughout this study were derived from HEK293F Viral Production Cells 2.0 (Gibco, Waltham, MA, USA), and cell culture supernatants were obtained from the Harcum lab (Department of Bioengineering, Clemson University). Cells were cultured in shake flasks, and collected fractions were centrifuged at 700 × *g* for 5 minutes to remove cells. The supernatant was filtered through a 0.2 µm PES filter to remove cell debris. The clarified culture medium (CCM) was aliquoted into 2 mL tubes and stored in -80 °C until use. The EV concentration in the stock sample was determined to be ~1.48 × 10^11^ particles mL^-1^.

### Assembly of polyester capillary-channeled polymer (C-CP) fiber columns

Trilobal PET C-CP fiber microbore columns were prepared in-house with slight modifications from our previously described method 28, 29. Briefly, eight rotations of fibers (528 individual fibers) were pulled through a 0.76-mm internal diameter (I.D.) PEEK tubing (IDEX Health & Science LLC, Oak Harbor, WA, USA) and trimmed to 30 cm in length. The 15 cm microbore columns were prepared similarly and then cut in half. Prior to use, columns were flushed for 10 minutes with 100% MP-C, then with 100% MP-D for 10 minutes, and equilibrated for 5 minutes with 100% MP-A. Columns were stored at room temperature until further use. For the EV isolation for downstream characterization, PET fibers were assembled in a 15 cm long analytical scale column having a 2.1 mm internal diameter (Chrom Tech, Apple Valley, MN, USA) with 40 fiber rotations. For optimal recovery, after appreciable decrease in peak area (typically 12 injections), fibers were flushed out of the column with water by applying a very high flow rate (6 - 8 mL min^-1^) while keeping the column outlet open (i.e., without the frit and frit holder) and new fibers placed in the column. Additionally, to assess the reproducibility and run-to-run repeatability of the column assembly, the fibers were flushed out after each set of successive injections, and replaced with new fibers.

### Nanoflow cytometry

Isolated fractions from HIC separation were characterized using a NanoAnalyzer instrument (NanoFCM Inc., Nottingham, UK) equipped with dual 488/638 nm lasers for simultaneous side scatter (SSC) and fluorescence detection. Bandpass filters (488/10 nm, 525/40 nm, and 670/30 nm) were used for signal collection in the respective channels. Instrument alignment and size/number density calibration were performed according to the manufacturer's instructions prior to analysis. Fluorescent silica microspheres (0.25 µm, 2.17 × 10¹⁰ particles mL^-1^) were diluted 1:100 and used to align optics and lasers, and to standardize particle concentration. A silica nanosphere cocktail (SM16-Exo, 68-155 nm) was used to generate a size calibration curve. For fluorescence labeling, samples were diluted in 50mM phosphate buffer and incubated with MemGlow 640 dye (1:2500; Cytoskeleton Inc., USA) for membrane labeling and ExoBrite CD9 and CD81 antibodies (1:4000; Biotium, USA) for tetraspanin detection. Incubation was performed at 37 °C for 3 hours in a water bath. The sample delivery pressure was maintained above 160 kPa, and the sampling pressure was set to 1 kPa. The system was flushed with the manufacturer's recommended cleaning solution, and the external capillary was rinsed by dipping in water between samples. Sample data sets were acquired for 30 seconds. nFCM Profession V2.0 software was used for system control and data processing.

### Nanoparticle tracking analysis

Nanoparticle tracking analysis (NTA) was used to determine the size distribution of the fractions and operated according to manufacturer's instructions. The Malvern NanoSight NS300 (Malvern Panalytical, Great Malvern, UK), controlled by the NanoSight NTA 3.4.4 software, was used for all measurements. The camera (sCMOS) and detection settings were not altered unless necessary; however, minor adjustments were made to minimize background noise. The camera level was maintained at 13-14, and the frame rate at 25 frames sec^-1^. Measurements were taken at ambient temperature, with the sample viscosity set by the software automatically. Samples were loaded into the flow cell with flow rate = 1000 (instrument units) for 20 seconds, followed by a flow rate = 100 for 120 seconds. Data acquisition proceeded with a 60-second imaging time, at flow rate = 100, with five replicates. Between each sample, the flow cell was purged first with DI H2O, then 10% IPA, for 2 minutes each with a flow rate of 1000. A syringe filled with air (~3 mL air) was then gently pushed into the inlet line to remove liquid from the flow cell. The ideal detection threshold was set on a sample-to-sample basis, with a debris count (blue cross count) less than 5, and a particle count greater than 30 per frame. This value varied from a detection threshold of 5 to 10. The blur size and max jump distance were controlled automatically.

### Transmission electron microscopy

TEM imaging was employed to verify the sizing and physical structure of the EV isolates. Each whole peak fraction (indicated in Fig. 2A) was buffer exchanged to 20 mM HEPES buffer, pH 7.0, and 10 µL of each whole peak fraction was placed on parafilm. Next, 10 µL of 1% uranyl acetate was pipetted onto the sample on the parafilm and mixed by pipetting up and down. The Carbon grid was then placed on the sample, facing down, and allowed to incubate for 15 s. The excess sample was blotted using lint-free paper. Grids were placed in a desiccator overnight and then imaged using a JEOL instrument with a 200-ms exposure time, an NS15 camera, and an accelerating voltage of 120 kV.

## Results

### Development of alternative gradient methods to isolate subpopulations of EVs

**Figure 1:**
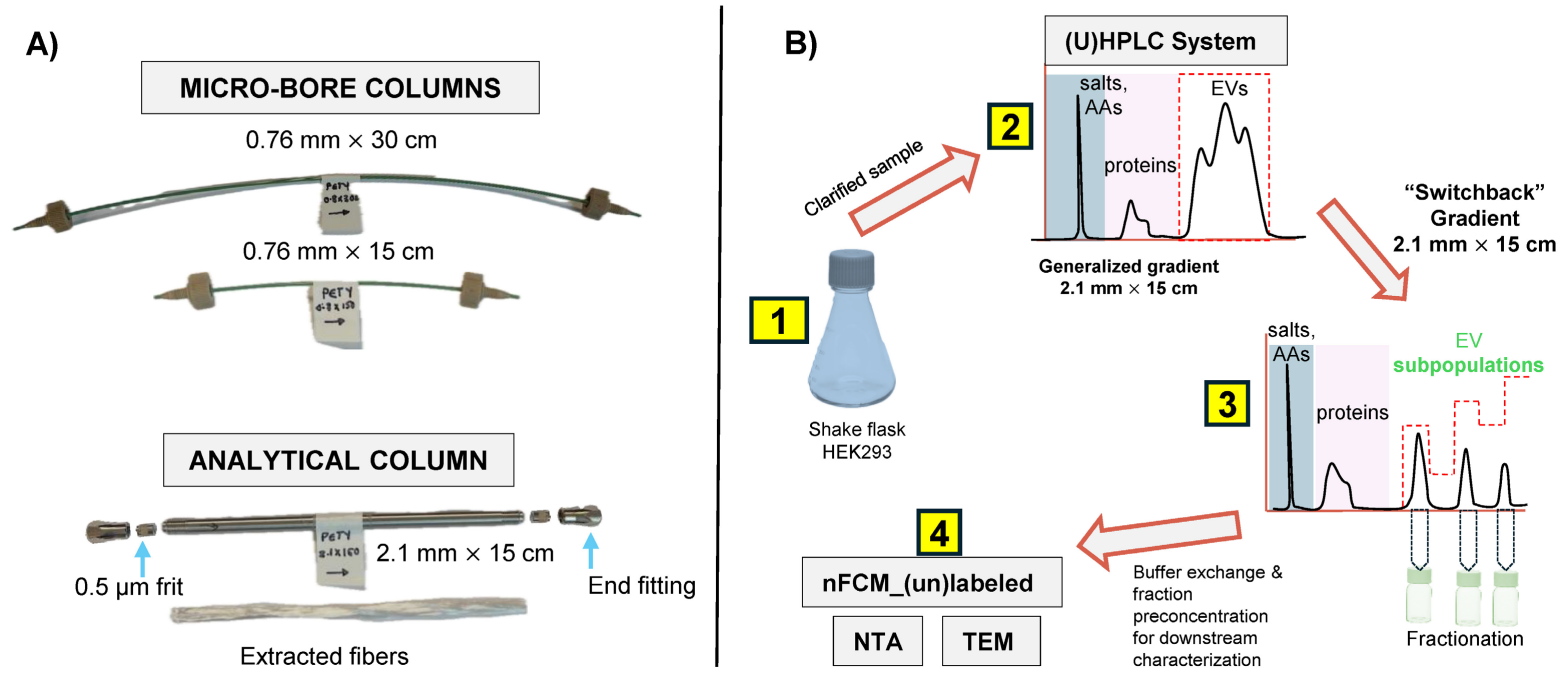
** A**) PET fiber columns in different formats. The microbore columns (0.76 mm I.D.) were used during the initial scouting runs, with the longer column used to achieve higher peak capacity. Capillary columns were cut to the desired length using a hard razor. The optimized separation method was applied to the analytical-scale column (2.1 mm I.D.) for sample enrichment prior to downstream characterization. A representative bundle of fibers extracted from the column is also shown. **B**) The overall experimental workflow applied in this study is summarized. AAs = amino acids, ccm = clarified culture media.

**Figure 2:**
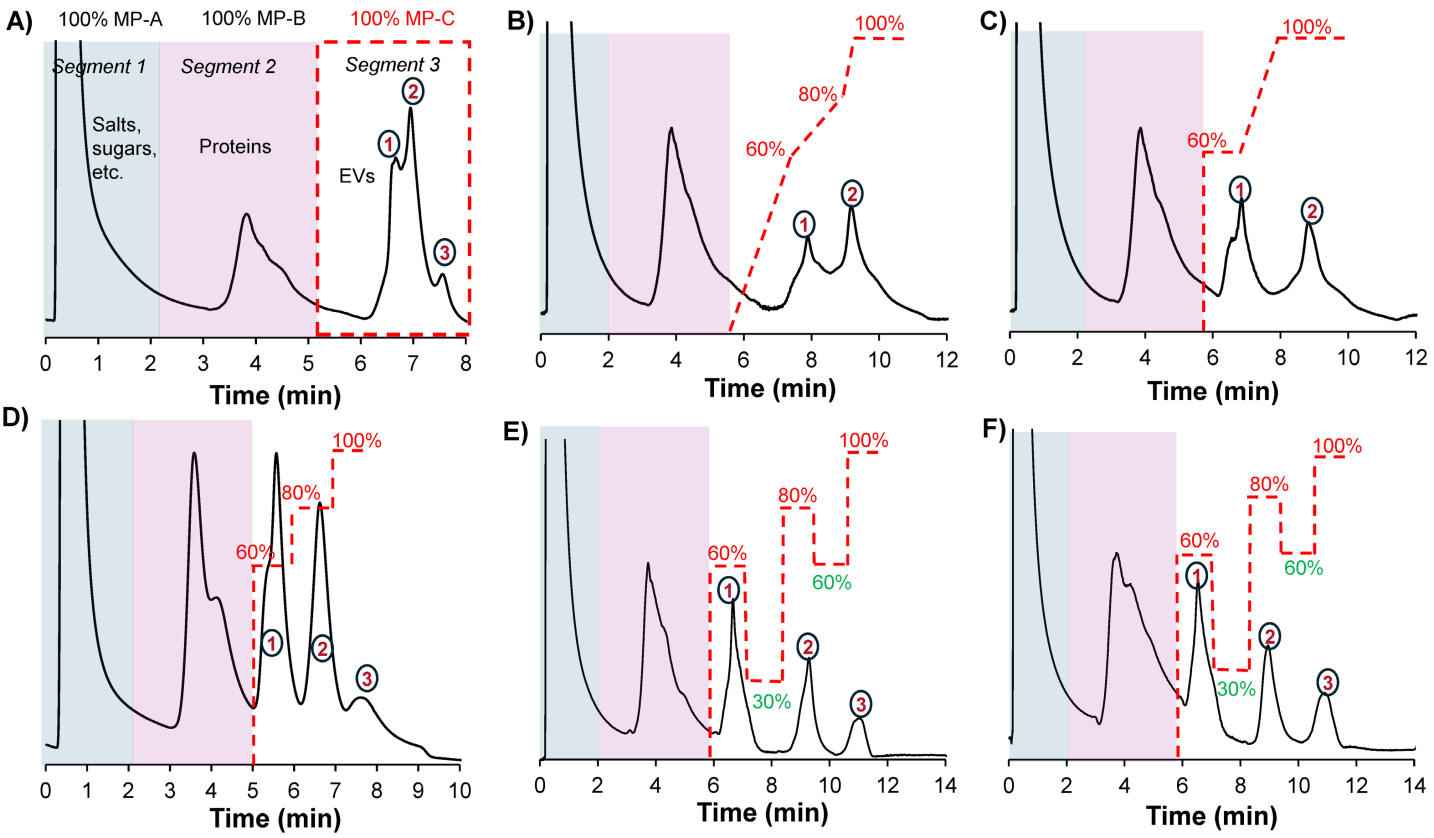
** A**) Chromatographic profile obtained when applying a generic gradient on a 0.76 mm x 15 cm column. Scouting gradient runs on a 0.76 mm x 30 cm column showing chromatographic peak profiles when applying **B**) a multilinear gradient, **C**) a combination of isocratic step gradients and a linear gradient and **D**) a multi-isocratic step gradient. The application of multi-isocratic step gradients with both positive and negative steps enabled the high-resolution separation of EV subpopulations on **E**) a 30 cm and **F**) 15 cm column. The flow rate was set to 0.5 mL min^-1^, and MP-D was delivered in-line with MP-C to modulate its effective composition during the run; for example, a composition of 60% MP-C included 40% MP-D. Gradient lines are guides for the eye and not drawn to scale.

The different column formats and a flow chart highlighting the general experimental steps used in this study are depicted in Figs. 1A and B, respectively. In a recent study, we demonstrated the repeatability of the 15 cm long x 0.76 mm I.D. polyester (PET) capillary-channeled polymer (C-CP) fiber microbore columns investigated towards the routine analysis of whole populations of HEK293-derived EVs, yielding < 0.6% RSD for retention time and < 3% RSD peak area across 10 columns from five batches. Figure 2A shows the chromatographic profile obtained when using the microbore column format and applying a generic step gradient consisting of three segments 25. In Segment 1, 100% mobile phase A (MP-A) is applied for 2 minutes to capture proteins and EVs, while media components, such as amino acids, sugars, and vitamins, elute with the injection volume. Next, 100% MP-B is applied for 3 minutes (Segment 2) to elute retained culture-derived proteins. Finally, 100% MP-C is applied for 3 minutes (Segment 3) to elute the target EVs. Interestingly, the EVs eluted as a broad peak with shoulders, suggesting the presence of partially resolved subpopulations having similar retention characteristics.

To improve the resolution, and intending to fully resolve these peaks, a systematic approach involving scouting runs with modifications in Segment 3 was performed, using MP-D (see the Method section for details) to modify the gradient composition. Multistep linear gradients, isocratic step gradients and a combination of both were scouted. Additionally, the column length was increased to 30 cm to increase the peak capacity. Figures 2B and C illustrate the elution profiles obtained when applying a multistep linear gradient and the combination of isocratic step gradients and a multistep linear gradient. With the multilinear gradients, a slight improvement in resolution is observed between the first two peaks as evidenced by the better-defined valley between the two peaks, but the anticipated resolution obtained for the third is not realized (Fig. 2B). When using a combination of isocratic step gradients in combination with a linear gradient, peaks 1 & 2 are almost baseline separated; however, the third peak is still not apparent (Fig. 2C). Generally, it was observed that the isocratic step gradients performed better than linear gradients. Our previous results demonstrated that linear gradient elution programs lead to prolonged analysis times and suboptimal recovery of the target EVs 30. This could potentially be due to the hydrophobicity of the stationary phase, which necessitates the use of step gradients with isocratic holds at high elution strengths to achieve the efficient desorption of the retained target vesicles. Therefore, only isocratic step gradients were used in subsequent scouting experiments.

When applying multistep isocratic gradients in Segment 3, comprising of 60%, 80%, and 100% MP-C for 1 min each, a significant improvement in the separation was observed, with the third peak becoming more apparent (Fig. 2D). To further improve the separation with the aim of achieving baseline resolution, the concept of a negative gradient, as has been demonstrated for closely-eluting proteins under RPLC conditions 31, was applied. By introducing a negative gradient step (*i*.*e*., 30% MP-C) after eluting the first peak with the 60%, the second peak is retained relatively longer and then eluted with the 80% step. Similarly, by introducing a negative gradient step (*i*.*e*., 60% MP-C), elution of the last peak is delayed and then eluted at 100% MP-C. This resulted in a high-resolution separation of the three fractions (Fig. 2E). Note that the mobile-phase compositions were empirically determined. The optimized gradient was also tested on the 15 cm column, yielding the same separation profile (Fig. 2F). We term this general approach of positive and negative gradient steps a “switchback gradient”.

### Verification of the quality and reproducibility of switchback HIC gradients on analytical-scale C-CP fiber columns

**Figure 3:**
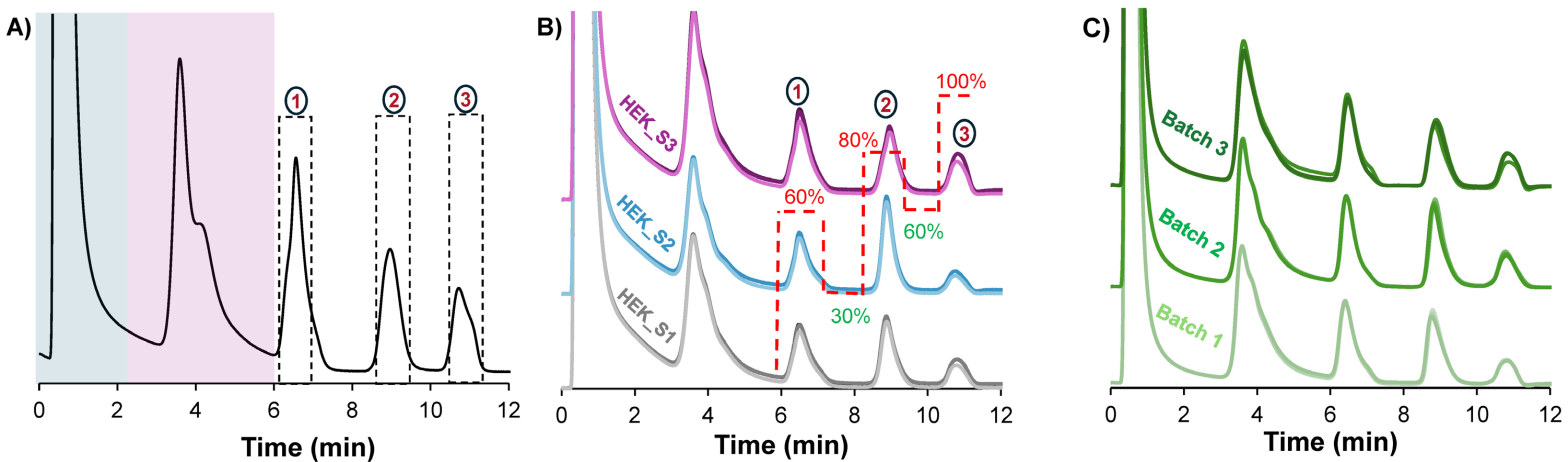
** A**) The resulting chromatographic peak profile, obtained by applying the high-resolution switchback method on a 2.1 mm x 15 cm column. **B**) An overlay of chromograms from triplicate injections of three different HEK293 ccm samples shows reproducibility across different samples. **C**) Reproducibility of column assembly is also demonstrated on three different columns with an overlay of triplicate injections of the same sample per column. The flow rate was maintained at 0.75 mL/min. The same method was applied in **A**, **B**, and **C**, but is only shown in **B** for clarity. Dotted lines in (**A**) represent the peak collection window.

Microbore HPLC columns are beneficial because they enable high sensitivity with reduced consumption of sample and mobile phase.^32^ However, they are limited by their mass/volume loading capacities, making them unsuitable when targeting downstream characterization where sample enrichment is required. In order to gain greater EV loading capabilities, fibers were assembled in 2.1 mm x 15 cm format analytical-scale columns. To increase the mass loading, the clarified HEK293 sample was preconcentrated to four times its original concentration using the Amicon 10 kDa MWC filters. With the preconcentrated sample, the maximum injection volume to maintain separation efficiency was empirically determined to be 2 μL. Beyond this volume, characteristic nonlinear features of column overload were observed. Figure 3A shows the chromatographic peak profile obtained using the optimized conditions for the larger column size. Clearly, the chromatographic program was transferable between the fiber column formats. For a qualitative assessment of the method stability and reproducibility, three different HEK293 samples were injected onto a column. These three samples originated from the same cell bank, but were cultured and harvested under slightly different conditions, and stored at -80 °C as the basis for a separate study regarding culture yields. An overlay of triplicate injections from each of the three samples (see Fig. 3B) shows similar chromatographic profiles, hence the method is stable and reproducible under the tested conditions.

Additionally, the method repeatability and column batch-to-batch reproducibility was assessed by performing five replicate injections on each of the three different PET C-CP fiber columns. Again, very similar peak profiles were observed across all three fiber columns as visualized in Fig. 3C. For a quantitative assessment, the retention time, peak area and peak width-at-half-height of the second EV peak were used as performance metrics, with the resulting descriptive statistics summarized in Table 1. Excellent method repeatability was observed across the triplicate columns, with RSD values under 0.3% for retention time and below 7% for peak area and peak width within each column. With respect to column batch variability, the very slight difference (1.2%) in retention time across the three columns indicates a high level of consistency in retention time and strong robustness and intermediate precision. For the peak area and peak width values, a wider variation is observed, 17% and 25% respectively, which is not surprising when considering the relatively simplistic manual assembly of the fiber columns. Nonetheless, the values observed within each column batch are quite acceptable for this novel isolation from a very complex sample. Most importantly, in every instance, the complete temporal isolation of the three subpopulations is realized with ease.

### Multi-modal characterization and confirmation of distinct EV size subpopulations

**Figure 4:**
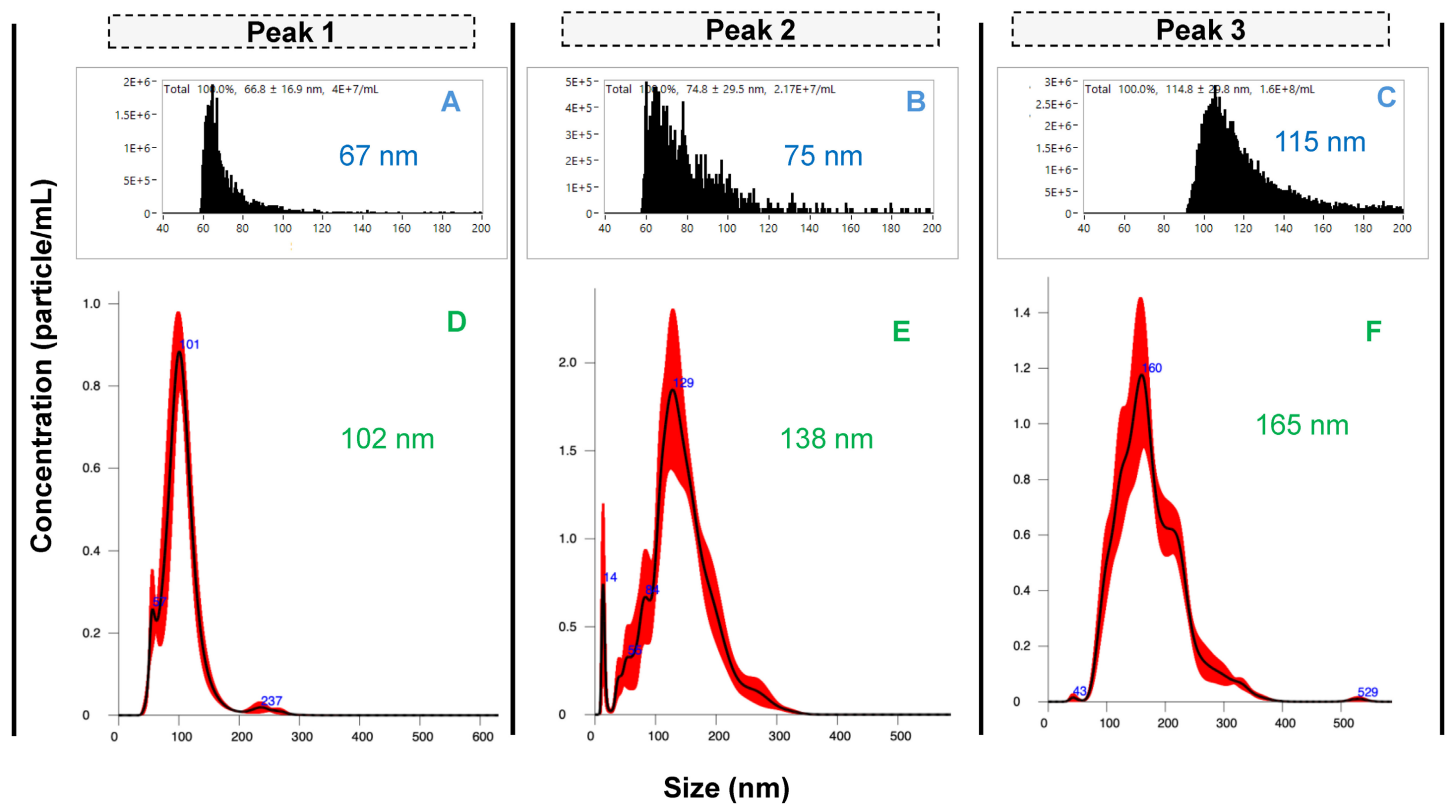
Size characterization of EV peaks using **A-C**) nanoflow cytometry and **D-F**) nanoparticle tracking analysis. Peaks 1 - 3 were pooled from 40 injections.

As described previously, the existence of subpopulations of EV sizes and content is a central focus in the basic biochemistry of the vesicles 8, 10. To assess the potential physical differences between the Segment 3 peak isolates and to confirm the presence of EVs in each peak, eluates were collected for each peak using the automated integral fraction collector, see highlighted regions on Fig. 3A. Fractions were pooled across 40 injections, buffer-exchanged to 50 mM phosphate buffer at 4500 rpm, 15 min at 8°C using 10 kDa Amicon filters (3 times) and then preconcentrated to a final volume of 1 mL. For comprehensive characterization, each fraction was first analyzed using nanoflow cytometry (nFCM), which revealed distinct size distributions for the fractions corresponding to peaks 1, 2, and 3, yielding median sizes ± SD of 67 ± 17 nm, 75 ± 30 nm, and 115 ± 30 nm, respectively (Figs. 4A-C). The size distribution corresponding to peak 1 is relatively narrow in comparison to peaks 2 and 3, with less skewing towards larger particle sizes as well. Peaks 1 and 2 have a similar size range (60-100 nm), although peak 2 has a relatively higher population between 80 and 100 nm. On the contrary, peak 3 reveals populations in the higher size range (~90 - 140 nm), with a significant population between 100 and 120 nm.

The differences in the size distributions among the fractions were further confirmed by NTA, a benchmark method in the realm of EV characterization 33, 34. The product scattering data are presented in Figs. 4D-F, with the size ranges observed being in very good agreement between the nFCM and NTA, both in the ranges for small EVs (30 - 200 nm). The particle sizes determined by NTA increased across the respective elution fractions, with median size ± SD of 102 ± 32 nm, 138 ± 50 nm, and 165 ± 57 nm for peaks 1, 2 and 3, respectively. The difference in sizes between the particles observed by NTA are approximately greater by 45 nm than the respective sizes observed with nFCM. While there are reasons for the differences in the absolute values, three distinct size populations are clearly identified.

**Figure 5:**
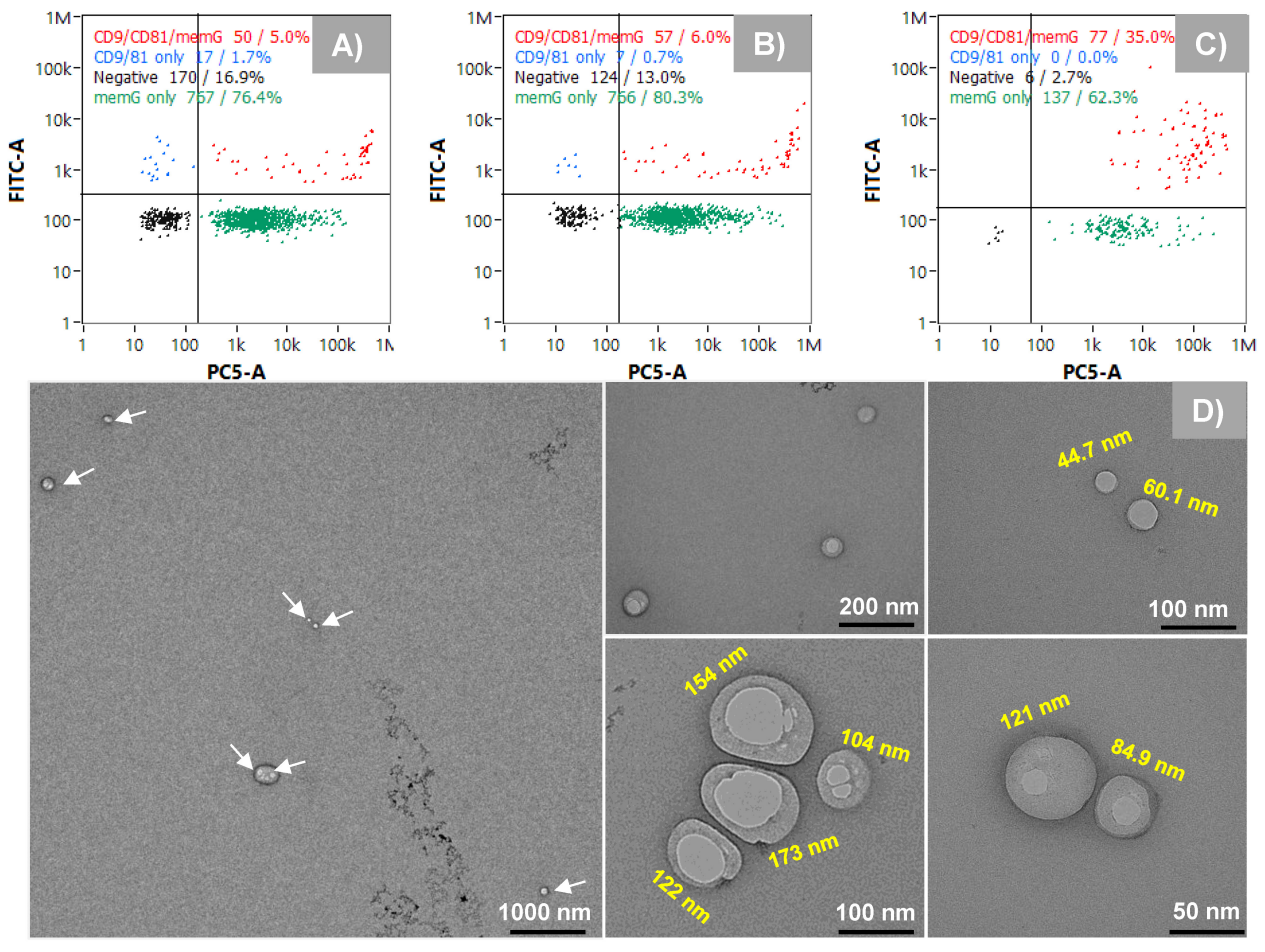
**A-C**) Confirming the presence of EVs in isolated peak fractions using surface markers (CD9 and CD81) as probes and the bilayer membrane (MemGlow). **D**) Transmission electron microscopy (TEM) images showing vesicles with intact membranes.

While the nFCM and NTA data clearly point to three distinct particle size populations, any approach to size select EVs must also confirm that those particles are indeed the intended products. To further assess the identity of the recovered particles as EVs, the fluorescence capabilities of the nanoFCM were employed to confirm the presence of the tetraspanin surface markers CD9 and CD81, probed with FITC-labeled anti-CD9 and anti-CD81 antibodies. In addition, membrane labeling with MemGlow confirmed the presence of vesicles having the characteristic lipid bilayer membranes. The representative quad-plots of Figs. 5A-C present the case wherein each of the collected fractions were double-positive for the tetraspanin probes and the membrane dye. As expected, the generic membrane probe coupling was more effective than the labelled antibodies as the target proteins exist in very low numbers on each EV. Very interestingly, while fractions 1 and 2 show 5 and 6% double-positive responses respectively, fraction 3 was significantly higher, up to 35%. While this difference in response could reflect the larger size of the particles (more tretraspanins to be labeled), it may also reflect fundamental differences in surface protein composition among the subpopulations, as has been postulated 11, 15, 19. Since a cocktail of the FITC-labeled probes was used, the relative abundance between the individual tetraspanins across the different isolates cannot be inferred. This can be investigated using nFCM with individual probes or with single EV assays, such as direct stochastic optical reconstruction microscopy (dSTORM) 35. The final form of EV confirmation was through TEM. Due to the low sampling efficiency of TEM, the entire EV peak was collected under high-throughput conditions (see Fig. 2A, *Segment 3*, where the whole peak was collected between 6 and 8 min). Here, the TEM images shown in Fig. 5D reveal a wide size range (45 nm - 173 nm), reflective of all three populations, and having the expected intact membranes and cup-shaped structures. As such, the designation of the collected fractions as subpopulations of EVs in the exosome size range is supported. Certainly, far greater numbers of EVs would need to be included in the TEM analysis to provide statistically-significant size conclusions.

## Discussion

Conventional methods for isolating EVs mainly focus on recovering the bulk population of EVs, largely due to their low resolving power and the inherent heterogeneity of EVs. However, in liquid biopsy and biological function studies, for example, separating EV subpopulations has been found to provide critical insights 36. With regards to therapeutic application, a current focus in the field is to identify and unravel the molecular composition of the EV population responsible for a desired function. Achieving this requires either refining existing methods or developing high-resolution methods. For example, Willms *et al*. employed sucrose density gradient centrifugation to isolate subpopulations of exosomes with distinct molecular and biological properties 11. While this method was validated for different cell types, it is labor-intensive and time-consuming (requiring > 72h), limiting its suitability for routine use. More recently, Kim *et al*. described a method that separates exosomes and microvesicles using a combination of ultrafiltration (UF) and transverse flow field-flow fractionation (T4F) 37. Although the T4F separation is achieved under 40 min, the preceding UF is time-consuming and introduces another level of complexity. Moreover, these methods face major challenges with regards to scale-up. Here, we introduce a rapid and low-cost method for enriching distinct EV subpopulations, thereby opening new avenues for characterizing the molecular content and studying subtype-specific function of different EV subpopulations. Our method enables the separation of EV subpopulations, defined by differences in chromatographic behavior based on relative hydrophobicity, from HEK293 clarified cell culture media in a total run time of 16 minutes, using PET C-CP fiber columns in both microbore and analytical scale formats. The repeatability and intermediate precision of the method in analytical scale column format (2.1 I.D.) enables sample enrichment for downstream characterization. The columns can be assembled within 10 minutes. Notably, the column hardware can be used infinitely (*i.e.*, fibers can be replaced after appreciable decrease in respective peak area, typically after ~12 injections) which significantly reduces the overall cost of the column compared to packed bed HIC columns. To the best of our knowledge, there is yet a commercial column designed for HIC mode separation of EVs 38. This study represents the first chromatographic method demonstrating the clean resolution of EV subpopulations. Note that this method is demonstrated for the single case of EVs isolated from HEK293 culture media. Other matrices need to be investigated to confirm the general applicability of the method, and this will be evaluated in future studies.

Perhaps the most important observation is that the different subpopulations elute in order of increasing size, which may suggest a secondary separation mechanism based on hydrodynamic size, though this is the opposite of a size exclusion chromatography (SEC) mechanism. However, the PET fibers do not have macropores, which rules out this possibility 39. Theoretically, the number of membrane proteins, and thus vesicle hydrophobicity, increases relative to the EV surface area and volume. Therefore, we hypothesize that the elution trend is related to the surface hydrophobicity as reflected in the elution solvent compositions, where large EVs are more retained due to their relatively higher hydrophobicity. Moreover, the nFCM data shows that the first two peaks have closely similar population size range, thus these populations may likely differ more in surface composition rather than size. As such, the contribution of this mechanism cannot be completely ruled out.

Given the highly complex surface structure of EVs, it is unlikely that retention is driven by a single interaction mechanism. While hydrophobic interactions are expected to dominate under the experimental conditions used here, additional contributions such as electrostatic interactions and lipid-mediated van der Waals forces may influence retention behavior. Quantitative structure-retention relationships (QSRR) could be used to probe the separation mechanism by identifying the most informative structural descriptors and physicochemical properties associated with the different EV subpopulations 40. A detailed mechanistic investigation of these contributions, however, is beyond the scope of this study. Nonetheless, comprehensive multi-omics analyses (lipidomics, proteomics, and glycomics) will be valuable for elucidating molecular differences between the subpopulations and will be the focus of future studies.

The existence of discrete-sized populations among the three fractions was verified via two independent mechanisms employing nFCM and NTA. The discrepancy in sizes between nFCM data and NTA may likely be due to a difference in detection mechanism or a difference in the sensitivity of the instruments. While nFCM requires < 10^8^ particles mL^-1^ for optimum sensitivity, NTA requires > 10^8^ particles mL^-1^ as both methods had been successfully validated based on manufacturer's guidance. Since the concentrations of the fractions were < 10^7^ particles mL^-1^ (nFCM data), the NTA data might be slightly biased, resulting in the inclusion of sample artifacts and consequently inflating the true population size. We have observed a similar discrepancy in sizing between the two instruments, even for relatively “pure” samples such as silica nanoparticles 41. Moreover, size discrepancies have also been reported for the same sample when using NTA instruments from different manufacturers 34. This is a general bottleneck in the field: the lack of standardized reference materials and corresponding characterization methods 42.

## Conclusions

Interest in extracellular vesicles has increased significantly over the last decade, with key advances across both fundamental and applied research. The potential of these bio-nanoparticle biomarkers and therapeutic vectors for human health is becoming well established. Yet, current separation methods do not provide the high resolution needed to separate heterogeneous populations of EVs, especially in the smaller size range (<200 nm), which slows down identification of size populations responsible for a desired effect, and consequently their full-scale translation. Promising results have been obtained with density gradient centrifugation and a combination of UF and T4F. However, these methods are time-consuming and not suitable for high-throughput routine applications.

To this end, we have developed a switchback gradient modulation strategy that selectively elutes distinct exosomal subpopulations defined by differences in chromatographic behaviour predominantly through their surface hydrophobicity, demonstrating the high-resolving capabilities of polyester C-CP fiber columns. The method allows enrichment of exosome subpopulations from HEK293 clarified culture media within a total run time of 16 minutes. The potential for routine application has been demonstrated, with high reproducibility across different sample and column batches, addressing another bottleneck in the field. Studies underway have improved the column load by (i) increasing the fiber packing density, (ii) introducing a void at the column inlet, which helps in premixing. This has substantially increased the column load, allowing injection of 4 µL of a 4X preconcentrated sample. This is currently used to isolate fractions for multi-omics analyses.

Future efforts will look to increase sample loading and reduce the need for multiple-injection pooling, requiring continued investigation of columns of larger format. Current loading capcities of ~10^12^ EVs per column in <15 process times will need to be augmented with the capability to achieve high loadings while retaining subpopulation resolution. This capability (and at higher capacities) will prove invaluable in terms of the tailoring of vector production processes. Additionally, it would also be worthwhile to assess the reproducibility of the downstream characterization data (size) from multiple independent preparations. Nonetheless, with the current format, this fast, cost-effective approach would be critical for providing the samples needed for routine biochemistry studies, multi-omics profiling, and ultimately, supporting biomarker discovery.

## Figures and Tables

**Table 1:** Quantitative analysis showing data obtained from five successive injections (n = 5) on three fiber batches. Values are shown as Mean ± SD (%RSD), where SD is standard deviation and RSD is relative standard deviation. Peak @ w_1/2_ is peak width-at-half-height.

Metric	Retention time (min)	Peak area (mAU.min)	Peak @ *w_1/2_*
**Batch 1**	8.77 ± 0.01 (0.16 %)	13.25 ± 0.70 (5.3 %)	0.47 ± 0.03 (6.2 %)
**Batch 2**	8.82 ± 0.03 (0.32 %)	15.53 ± 0.80 (5.2 %)	0.42 ± 0.01 (2.7 %)
**Batch 3**	8.88 ± 0.02 (0.18 %)	13.61 ± 0.89 (6.5 %)	0.52 ± 0.03 (4.9 %)
**Global Mean**	8.82	14.13	0.47
**Variation**	1.20%	17%	25%

## Abbreviations

EVs: extracellular vesicles
HIC: hydrophobic interaction chromatography
C-CP: capillary-channeled polymer
NTA: nanoparticle tracking analysis nano-flow cytometry (nFCM)
TEM: transmission electron microscopy
HEK293: Human embryonic kidney-293
I.D.: internal diameter
MP: mobile phase
(U)HPLC: (Ultra)high performance liquid chromatography
UF: ultrafiltration
T4F: transverse flow field-flow fractionation
SEC: size exclusion chromatography
MCW: molecular weight cut-off
ACN: acetonitrile
PES: polyethersulfone
PEEK: polyether ether ketone
CCM: clarified culture medium
SSC: simultaneous side scatter
